# Comparison of Frontier Open-Source and Proprietary Large Language Models for Complex Diagnoses

**DOI:** 10.1001/jamahealthforum.2025.0040

**Published:** 2025-03-14

**Authors:** Thomas A. Buckley, Byron Crowe, Raja-Elie E. Abdulnour, Adam Rodman, Arjun K. Manrai

**Affiliations:** 1 Department of Biomedical Informatics, Harvard Medical School, Boston, Massachusetts; 2 Department of Medicine, Beth Israel Deaconess Medical Center, Boston, Massachusetts; 3 Division of Pulmonary and Critical Care Medicine, Brigham and Women’s Hospital, Boston, Massachusetts

## Abstract

This comparative effectiveness research assesses the performance of newer open-source large language models (LLMs) with that of closed-source proprietary large LLMs.

## Introduction

Large language models (LLMs) now perform cognitive tasks in medicine previously believed to be the sole domain of humans, including accurately answering medical multiple-choice questions,^1^ demonstrating nuanced clinical reasoning,^2^ and establishing a robust differential diagnosis for complex diagnostic cases.^3^ Since its release in March 2023, the GPT-4 (Generative Pre-trained Transformer 4) model (hereafter, *the closed-source LLM*) created by OpenAI has been among the best-performing LLMs on medical tasks and is being incorporated into health care applications. Although open-source LLMs have been available, they have generally not performed as well as proprietary models.^4^ However, it remains unclear how newer open-source models, such as the 405-billion parameter Llama 3.1 model (Meta) (hereafter, *the open-source LLM*), perform. Such open-source frontier models, named for their superior performance on benchmarks, may now be competitive alternatives to closed-source models.

## Methods

We evaluated the open-source LLM from August 6 to August 10, 2024, on 70 challenging diagnostic cases used previously to assess the closed-source LLM (case set 1).^3^ To mitigate risk of memorization, we retrieved 22 cases published between January 2024 and July 2024 (case set 2), after pretraining of the open-source LLM (December 2023). Cases come from the case records of the Massachusetts General Hospital series published by the *New England Journal of Medicine*; we obtained written permission from the Massachusetts Medical Society to use these cases. The models were not permitted to search the internet. The prompt is in the eAppendix in Supplement 1. A quality score^5^ was assigned to outputs independently by B.C. and A.R.; discordance was measured by linear-weighted Cohen κ, and discordant scores were reconciled through discussion. *P* values were computed using the 2-sided McNemar test and deemed statistically significant at *P* < .05. Analysis was performed in R, version 4.3.2. This study was deemed not human subjects research and did not require institutional review board oversight per the Common Rule. This study followed the STROBE reporting guideline.^6^

## Results

The reviewers agreed on 66% of differential quality scores (46 of 70; κ = 0.39) in the prior evaluation of the closed-source LLM and on 78% of cases (72 of 92; κ = 0.69) in this evaluation of the open-source LLM. We compared the distribution of differential diagnosis quality scores between the 2 models (Figure).^3,5^ In case set 1, the open-source LLM included the final diagnosis in the differential for 70% of cases (49 of 70) compared with 64% (45 of 70) for the closed-source LLM (*P* = .35). The first suggestion from the open-source LLM was correct for 41% of cases (29 of 70) compared with 37% (26 of 70) for the closed-source LLM. For case set 2, the open-source LLM included the final diagnosis in the differential in 73% of cases (16 of 22) and identified the final diagnosis as the first suggestion in 45% of cases (10 of 22). Example cases in which the models diverged in diagnostic quality are shown in the Table.

**Figure.**
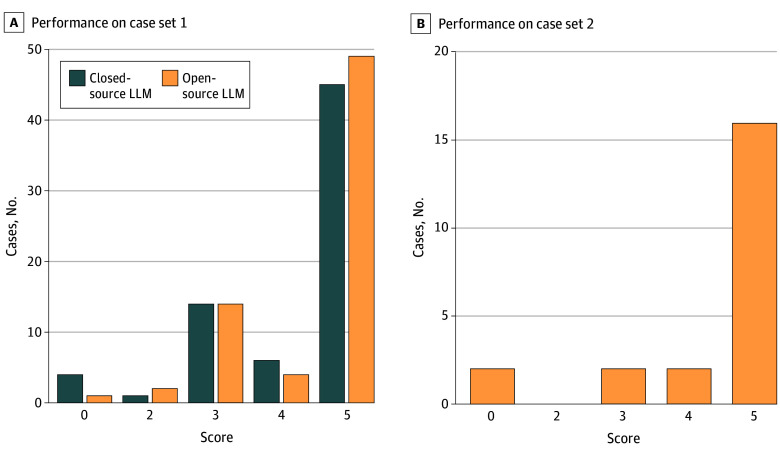
Performance of 2 Large Language Models (LLMs) on Complex Diagnostic Challenges Histogram comparison of differential diagnosis quality by the GPT-4 (Generative Pre-trained Transformer 4) model (closed-source LLM) and the 405-billion parameter Llama 3.1 model (open-source LLM) using the quality score metric on case set 1 (A) and case set 2 (B). The scoring used is a version adapted by Kanjee et al^3^ from the original in Bond et al^5^ (the original assigned inversely to the one presented here). The scale used is as follows: 5 = the actual diagnosis was suggested in the differential; 4 = the suggestions included something very close, but not exact; 3 = the suggestions included something closely related that might have been helpful; 2 = the suggestions included something related, but unlikely to be helpful; and 0 = no suggestions close to the target diagnosis.

**Table.** Example Cases Using Both LLMs

Final diagnosis	List of diagnoses	
	Closed-source LLM	Open-source LLM
**A. Case 26-2022: open-source LLM identifies correct diagnosis; closed-source LLM, incorrect**		
Langerhans cell histiocytosis of the lung and liver	Eosinophilic granulomatosis with polyangiitis Hypereosinophilic syndrome Eosinophilic pneumonia Parasitic infection (eg, strongyloidiasis or visceral larva migrans) Disseminated fungal infection (eg, histoplasmosis or coccidioidomycosis) Lymphoma with eosinophilic infiltration Metastatic cancer with eosinophilic infiltration Sarcoidosis with eosinophilic infiltration Drug-induced eosinophilic syndrome Quality score: 0	Langerhans cell histiocytosis Metastatic cancer (eg, breast, thyroid, or colon cancer) Sarcoidosis Tuberculosis Fungal infection (eg, histoplasmosis or cryptococcosis) Parasitic infection (eg, echinococcosis or toxocariasis) Granulomatosis with polyangiitis Erdheim-Chester disease Rosai-Dorfman disease Idiopathic hypereosinophilic syndrome Quality score: 5
**B. Case 4-2022: closed-source LLM identifies correct diagnosis; open-source LLM, incorrect**		
Neurosyphilis	Cogan syndrome Granulomatosis with polyangiitis (Wegener) Sarcoidosis Susac syndrome Behcet disease Vogt-Koyanagi-Harada syndrome Relapsing polychondritis Meniere disease Lyme disease Syphilis Autoimmune inner ear disease Acute viral labyrinthitis Quality score: 5	Cogan syndrome Sarcoidosis Wegener granulomatosis (granulomatosis with polyangiitis) Relapsing polychondritis Rheumatoid arthritis with ocular and auditory manifestations Lupus erythematosus with ocular and auditory manifestations Giant cell arteritis Polyarteritis nodosa Churg-Strauss syndrome (eosinophilic granulomatosis with polyangiitis) Endolymphatic sac tumor (unlikely, but possible given the hearing loss and vestibular symptoms) Quality score: 2

Abbreviations: closed-source LLM, Generative Pre-trained Transformer 4 model; LLM, large language model; open-source LLM, 405-billion parameter Llama 3.1 model.

## Discussion

For the first time, to our knowledge, an open-source LLM performed on par with GPT-4 in generating a differential diagnosis on complex diagnostic challenge cases. Our findings suggest an increasingly competitive landscape in LLM clinical decision support, and that institutions may be able to deploy high-performing custom models that run locally without sacrificing data privacy or flexibility.

This study has several limitations. First, details about the training data for LLMs are not publicly released. However, the open-source LLM exhibited similar performance on 22 cases published after pretraining ended. Second, these evaluations consider a single diagnostic task in which a highly informative summary of a clinical case is provided, which may not reflect the breadth of clinical reasoning. Future evaluations should use cases from clinical settings, including electronic health records. Third, this study had limited statistical power to assess differences between LLMs. Overall, the availability of an open-source LLM that performs on par with a leading proprietary model may both broaden access and expand possibilities of fine-tuning custom LLMs for integration in clinical care settings.

## Notes

Supplement 1.
eAppendix.

Supplement 2.
Data Sharing Statement
