# Supplementary material for: Comparison of Frontier Open-Source and Proprietary Large Language Models for Complex Diagnoses
Source: JAMA Health Forum. 2025 Mar 14;6(3):e250040. doi: 10.1001/jamahealthforum.2025.0040 (PMC11909604; doi:10.1001/jamahealthforum.2025.0040)
Supplement: Supplement 1. — eAppendix. [file jamahealthforum-e250040-s001.pdf]

# Supplementary Online Content

Buckley TA, Crowe B, Abdulnour REE, Rodman A, Manrai AK. Comparison of frontier open-source and proprietary large language models for complex diagnoses. *JAMA Health Forum.* 2025;6(3):e250040. doi:10.1001/jamahealthforum.2025.0040

**eAppendix.**

This supplementary material has been provided by the authors to give readers additional information about their work.

© 2025 Buckley TA et al. *JAMA Health Forum.*

**eAppendix.**

**Model Versions**
The Llama 3.1 405B model used is the pretrained meta-llama/Llama-3.1-405B-Instruct. GPT-4 was accessed using ChatGPT in the prior study (Kanjee et al. *JAMA* 2023).

**Model Prompts (same as used in Kanjee et al. *JAMA* 2023)**
I am running an experiment on a clinicopathological case conference to see how your diagnoses compare with those of human experts. I am going to give you part of a medical case. These have all been published in the New England Journal of Medicine. You are not trying to treat any patients. As you read the case, you will notice that there are expert discussants giving their thoughts. In this case, you are "Dr. {GPT-4, Llama}," an AI language model who is discussing the case along with human experts.

A clinicopathological case conference has several unspoken rules. The first is that there is most often a single definitive diagnosis (though rarely there may be more than one), and it is a diagnosis that is known today to exist in humans. The diagnosis is almost always confirmed by some sort of clinical pathology test or anatomic pathology test, though in rare cases when such a test does not exist for a diagnosis the diagnosis can instead be made using validated clinical criteria or very rarely just confirmed by expert opinion. You will be told at the end of the case description whether a diagnostic test/tests are being ordered, which you can assume will make the diagnosis/diagnoses.

After you read the case, I want you to give two pieces of information.

The first piece of information is your most likely diagnosis/diagnoses. You need to be as specific as possible -- the goal is to get the correct answer, not a broad category of answers. You do not need to explain your reasoning, just give the diagnosis/diagnoses.

The second piece of information is to give a robust differential diagnosis, ranked by their probability so that the most likely diagnosis is at the top, and the least likely is at the bottom. There is no limit to the number of diagnoses on your differential. You can give as many diagnoses as you think are reasonable. You do not need to explain your reasoning, just list the diagnoses. Again, the goal is to be as specific as possible with each of the diagnoses.

Do you have any questions, Dr. {GPT-4, Llama}?

Here is the case:

© 2025 Buckley TA et al. *JAMA Health Forum.*
