# Supplementary material for: Comparison of Frontier Open-Source and Proprietary Large Language Models for Complex Diagnoses
Source: JAMA Health Forum. 2025 Mar 14;6(3):e250040. doi: 10.1001/jamahealthforum.2025.0040 (PMC11909604; doi:10.1001/jamahealthforum.2025.0040)
Supplement: Supplement 2. — Data Sharing Statement [file jamahealthforum-e250040-s002.pdf]

Data Sharing Statement

Buckley. Comparison of Frontier Open-Source and Proprietary Large Language Models for Complex Diagnoses. *JAMA Health Forum*. Published March 14, 2025.
doi:10.1001/jamahealthforum.2025.0040

Data
Data available: Yes
Data types: Other (please specify)
Additional Information: Code for analysis.
How to access data: We will release the code used in this study with publication.
When available: With publication

Supporting Documents
Document types: Statistical/analytic code
How to access documents: We will release the code publicly on GitHub to all interested parties.
When available: With publication

Additional Information
Who can access the data: We will release the code publicly on GitHub to all interested parties.
Types of analyses: We will release the code publicly on GitHub to all interested parties.
Mechanisms of data availability: We will release the code publicly on GitHub to all interested parties.
